# Halogenated Indoles Decrease the Virulence of Vibrio campbellii in a Gnotobiotic Brine Shrimp Model

**DOI:** 10.1128/spectrum.02689-22

**Published:** 2022-09-26

**Authors:** Shanshan Zhang, Qian Yang, Tom Defoirdt

**Affiliations:** a Center for Microbial Ecology and Technology (CMET), Ghent University, Gent, Belgium; University of North Carolina at Chapel Hill

**Keywords:** antivirulence therapy, indole signaling, quorum sensing, shrimp, virulence

## Abstract

Indole signaling is viewed as a potential target for antivirulence therapy against antibiotic-resistant pathogens because of its link with the production of virulence factors. This study examined the antimicrobial and antivirulence properties of 44 indoles toward Vibrio campbellii. Based on the results, 17 halogenated indole analogues were selected, as they significantly improved the survival of brine shrimp larvae challenged with *V. campbellii*. Specifically, 6-bromoindole, 7-bromoindole, 4-fluoroindole, 5-iodoindole, and 7-iodoindole showed a high protective effect, improving the survival of brine shrimp to over 80% even at a low concentration of 10 μM. To explore the impact of selected indole analogues on bacterial virulence phenotypes, swimming motility, biofilm formation, protease activity, and hemolytic activity of *V. campbellii* were determined. The results showed that all of the 17 selected indole analogues decreased swimming motility at both 10 μM and 100 μM. Most of the indole analogues decreased biofilm formation at a concentration of 100 μM. In contrast, only a slightly decreased protease activity and no effect on hemolytic activity were observed at both concentrations. To our knowledge, this is the first study of the structure-activity relation of halogenated indole analogues with respect to virulence inhibition of a pathogenic bacterium in an *in vivo* host model system, and the results demonstrate the potential of these compounds in applications aiming at the protection of shrimp from vibriosis, a major disease in aquaculture.

**IMPORTANCE** Bacterial diseases are a major problem in the aquaculture industry. In order to counter this problem, farmers have been using antibiotics, and this has led to the evolution and spread of antibiotic resistance. In order for the aquaculture industry to further grow in a sustainable way, novel and sustainable methods to control diseases are needed. We previously reported that indole signaling is a valid target for the development of novel therapies to control disease caused by Vibrio campbellii and related bacteria, which are among the major bacterial pathogens in aquaculture. In the present study, we identified indole analogues that are more potent in protecting brine shrimp (a model organism for shrimp) from *V. campbellii*. To our knowledge, this is the first study of the structure-activity relation of halogenated indole analogues with respect to virulence inhibition of a pathogenic bacterium in an *in vivo* host model system.

## INTRODUCTION

Shrimp and prawns have historically been one of the most heavily traded fish products, and shrimp farming is one of the major parts of the aquaculture sector (1–3). However, bacterial diseases, such as vibriosis caused by various vibrios, are limiting the further sustainable expansion of aquaculture, especially in the early life stages of the animals (4–6). Vibrios belonging to the *Harveyi* clade are causing severe losses in shrimp farming, with up to 100% mortality in postlarvae and juveniles (7–9). Vibrio harveyi and Vibrio campbellii are closely related species belonging to the *Harveyi* clade. Both of them are important pathogens causing disease in wild and cultured aquatic organisms, including shrimp, fish, and molluscs, and this is leading to huge losses in the aquaculture industry worldwide (9, 10). In this situation, antibiotics often are the only effective agents that farmers have to protect their animals from bacterial infections. However, the wide and frequent use of antibiotics has resulted in the emergence of multidrug-resistant strains and has led to an international health crisis of both aquatic animals and humans (11, 12). Therefore, there is an urgent need for a truly novel strategy instead of antibiotics to control bacterial diseases in aquaculture (13). Antivirulence therapy is one of the newly developed therapeutic strategies; it aims at disarming pathogens by inhibiting their virulence rather than killing them. This strategy has been deemed to be a promising alternative strategy, as bacteria will probably have a lower tendency to develop resistance against it (14, 15).

Indole signaling is viewed as a potential target for antivirulence therapy against antibiotic-resistant pathogens because of its ability to inhibit other quorum-sensing (QS) systems and the production of virulence factors. Indole and indole analogues are widespread in the natural environment, as a variety of bacteria and some plants produce large quantities of indole, which can be structurally modified by some non-indole-producing prokaryotes and eukaryotes (16–18). Indole is an interspecies and intercellular signal; it attends diverse aspects of bacterial physiology, such as spore formation (19), plasmid stability (20), drug resistance (21, 22), biofilm formation (16, 23), and virulence (24, 25). Furthermore, indole and its aromatic heterocyclic structure are popularly used as synthetic starting points in the pharmaceutical industry (26), and some indole-based drugs are currently in clinical trials (27, 28). Recently, several indole analogues have been reported to reduce bacterial virulence. For example, 4-iodoindole, 7-iodoindole, 4-chloroindole, and 7-chloroindole were found to effectively inhibit biofilm formation, bacterial motility, fimbrial activity, hydrophobicity, protease activity, and indole production in Vibrio parahaemolyticus (29). Furthermore, 7-fluoroindole (30), 7-hydroxyindole (31), and 3-indolylacetonitrile (32) reduced the production of virulence factors in Pseudomonas aeruginosa. Finally, 5-fluoroindole, 6-fluoroindole, 5-methylindole, and 7-methylindole were proven to inhibit quorum sensing-controlled violacein pigment production in Chromobacterium violaceum CV026 and suppressed prodigiosin production, biofilm formation, swimming motility, and swarming motility in Serratia marcescens (33). In addition, halogenated indoles have been reported to have nematicidal and insecticidal potential (34, 35). Although the antivirulence activity of indole in *V. campbellii* has been reported in our previous work (23, 36), the concentration at which the best virulence-inhibitory activity occurred was also toxic to invertebrates.

**FIG 1:**
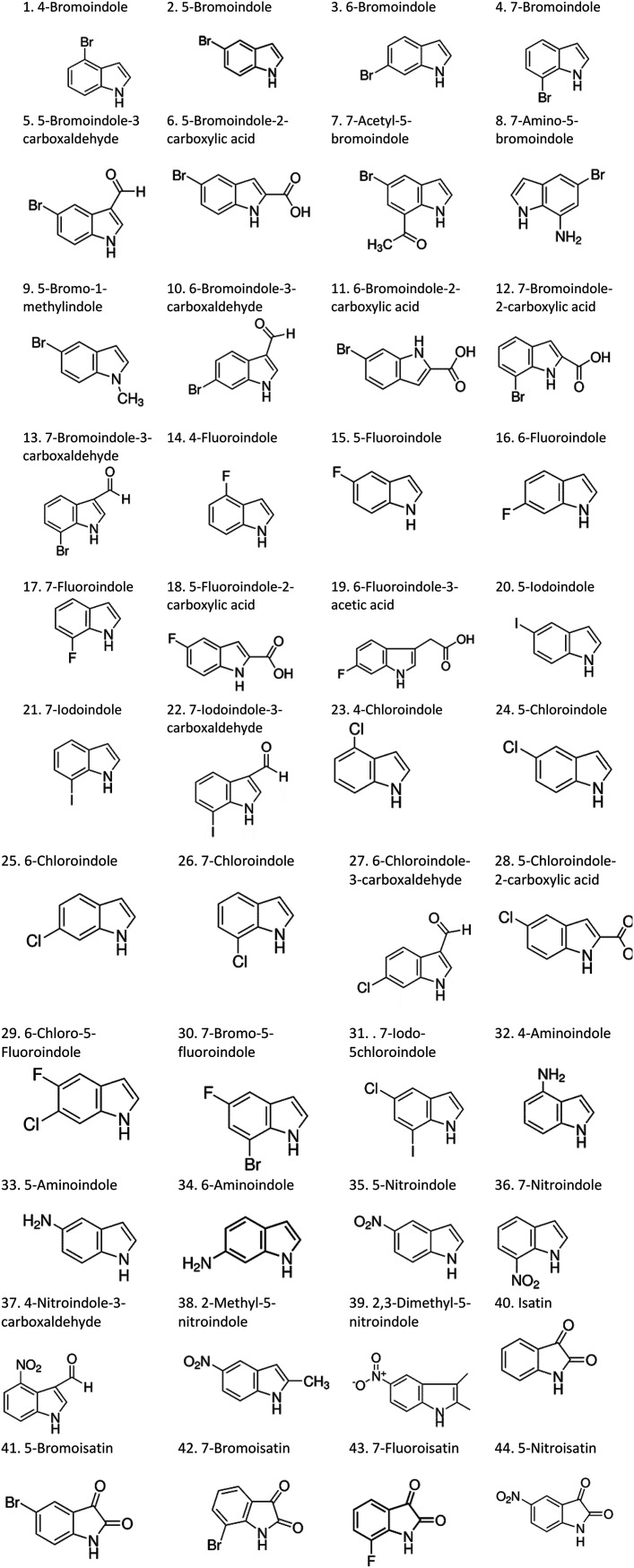
Structure of the indole analogues used in this study.

Therefore, the objectives of this study were to identify more potent indole analogues against *V. campbellii*, and to understand their structure-activity relationship. Initially, 44 indole analogues (Fig. 1) were investigated in terms of their abilities to protect brine shrimp larvae from infection by *V. campbellii*. Based on this test, 17 indoles were selected for further experiments aiming at testing their dose-response relations with respect to protecting brine shrimp larvae from vibriosis at lower concentrations and at verifying their impact on swimming motility, biofilm formation, protease activity, and hemolytic activity, which are major virulence factors of *V. campbellii*.

## RESULTS AND DISCUSSION

### Impact of the indole analogues on the growth of *V. campbellii*.

First, we determined the impact of the indole analogues on the growth of *V. campbellii*, as we aimed for analogues that affect the virulence but not the viability of the pathogen. Results showed that most of the indole analogues at a concentration of 200 μM did not affect bacterial growth, except for the isatins 40, 41, 42, 43, and 44 (see Fig. S1 in the supplemental material). Therefore, these compounds were excluded for further experiments.

### Impact of the indole analogues on the virulence of *V. campbellii* toward gnotobiotic brine shrimp (Artemia franciscana) larvae.

Based on the results of bacterial growth, indole analogues 1 to 39, which did not affect bacterial growth, were selected to explore the impact on the virulence of *V. campbellii* toward brine shrimp larvae. We added the indoles directly into the brine shrimp rearing water at a concentration of 20 μM together with *V. campbellii*. After 2 days of culture, an increased survival of brine shrimp larvae indicated the protective effect of several indole analogues. Results showed that 10 out of 13 brominated indoles, 4 out of 7 fluorinated indoles, 3 out of 3 iodinated indoles, 5 out of 6 chlorinated indoles, 1 out of 3 multiple halogenated indoles, 2 out of 3 aminoindoles, and 3 out of 5 nitroindoles improved the survival of challenged brine shrimp larvae (Table 1). Among these, 17 indole analogues increased the survival rate of challenged brine shrimp larvae to over 60%, including 7 brominated indoles, 2 fluorinated indoles, 3 iodinated indoles, 4 chlorinated indoles, and 1 multiple halogenated indole. Importantly, the protection offered by the compounds was obtained at a concentration (20 μM) that was well below the concentration that could inhibit the growth of *V. campbellii*, as there was no impact on growth up to 200 μM (Fig. S1). Hence, the MIC of the compounds is above 200 μM, whereas the virulence inhibitory concentration is 20 μM or lower.

**TABLE 1:** Percent survival of brine shrimp (Artemia franciscana) larvae after 2 days of challenge with *V. campbellii* and 20 μM indole analogues^*a*^

Experiment and treatment	Survival (%)	*P* value summary^*b*^	Live cell density (×10^5^ CFU/mL)
Expt 1			
Unchallenged shrimp larvae	87 ± 6		0
*V. campbellii*	2 ± 2		238 ± 61
*V. campbellii *+ 4-bromoindole	79 ± 12	***	203 ± 69
*V. campbellii *+ 5-bromoindole	73 ± 7	***	230 ± 85
*V. campbellii *+ 6-bromoindole	63 ± 17	***	163 ± 46
*V. campbellii *+ 7-bromoindole	82 ± 12	***	197 ± 45
*V. campbellii *+ 5-bromoindole-3-carboxaldehyde	50 ± 0	***	207 ± 56
*V. campbellii *+ 5-bromoindole-2-carboxylic acid	2 ± 2		213 ± 63
*V. campbellii *+ 7-acetyl-5-bromoindole	10 ± 7		158 ± 55
*V. campbellii *+ 7-amino-5-bromoindole	69 ± 7	***	222 ± 102
*V. campbellii *+ 5-bromo-1-methylindole	20 ± 9	*	212 ± 31
*V. campbellii *+ 6-bromoindole-3-carboxaldehyde	88 ± 10	***	178 ± 12
*V. campbellii *+ 6-bromoindole-2-carboxylic acid	8 ± 7		178 ± 42
*V. campbellii *+ 7-bromoindole-2-carboxylic acid	19 ± 4	*	188 ± 60
*V. campbellii *+ 7-bromoindole-3-carboxaldehyde	86 ± 14	***	165 ± 49
Expt 2			
Unchallenged shrimp larvae	100 ± 0		0
*V. campbellii*	6 ± 5		228 ± 38
*V. campbellii *+ 4-fluoroindole	96 ± 2	***	180 ± 41
*V. campbellii *+ 5-fluoroindole	31 ± 2	***	185 ± 62
*V. campbellii *+ 6-fluoroindole	53 ± 12	***	162 ± 60
*V. campbellii *+ 7-fluoroindole	82 ± 7	***	160 ± 64
*V. campbellii *+ 5-fluoroindole-2-carboxylic acid	4 ± 2		172 ± 29
*V. campbellii *+ 6-fluoroindole-3-acetic acid	7 ± 3		187 ± 58
*V. campbellii *+ 5-iodoindole	73 ± 12	***	182 ± 10
*V. campbellii *+ 7-iodoindole	68 ± 11	***	187 ± 50
*V. campbellii *+ 7-iodoindole-3-carboxaldehyde	81 ± 4	***	197 ± 13
*V. campbellii *+ 4-chloroindole	90 ± 0	***	203 ± 20
*V. campbellii *+ 5-chloroindole	82 ± 8	***	180 ± 68
*V. campbellii *+ 6-chloroindole	68 ± 15	***	170 ± 65
Expt 3			
Unchallenged shrimp larvae	96 ± 2		0
*V. campbellii*	0 ± 0		243 ± 50
*V. campbellii *+ 7-chloroindole	82 ± 5	***	235 ± 46
*V. campbellii *+ 6-chloroindole-3-carboxaldehyde	41 ± 28	***	215 ± 36
*V. campbellii *+ 5-chloroindole-2-carboxylic acid	6 ± 5		190 ± 13
*V. campbellii *+ 6-chloro-5-fluoroindole	83 ± 7	***	172 ± 35
*V. campbellii *+ 7-bromo-5-fluoroindole	17 ± 3		235 ± 46
*V. campbellii *+ 7-iodo-5-chloroindole	4 ± 2		222 ± 28
*V. campbellii *+ 4-aminoindole	32 ± 8	**	262 ± 21
*V. campbellii *+ 5-aminoindole	18 ± 5		148 ± 15
*V. campbellii *+ 6-aminoindole	21 + 17	*	320 ± 84
*V. campbellii *+ 5-nitroindole	12 ± 10		178 ± 62t
*V. campbellii *+ 7-nitroindole	47 ± 30	***	248 ± 31
*V. campbellii *+ 4-nitroindole-3-carboxaldehyde	11 ± 4		242 ± 40
*V. campbellii *+ 2-methyl-5-nitroindole	42 ± 15	***	205 ± 35
*V. campbellii *+ 2,3-dimethyl-5-nitroindole	50 ± 10	***	213 ± 28

a Average ± standard deviation of three shrimp cultures. Density of *V. campbellii* with or without 20 μM indole analogues in the brine shrimp larvae-rearing water after 2 days of challenge.

b *, Significantly different from treatment with unchallenged shrimp larvae (*P* ≤ 0.05); **, significantly different from treatment with unchallenged shrimp larvae (*P* ≤ 0.01); ***, significantly different from treatment with unchallenged shrimp larvae (*P* ≤ 0.001).

Although some halogenated indole analogues have been shown to affect quorum sensing and virulence factor production in various kinds of pathogens (Table 2), none of them have been tested *in vivo* in animals challenged with a pathogen of interest before. In the present study, we found that within the brominated, fluorinated, and chlorinated indoles, in general, the 4- and 7-substituted compounds showed the highest activity, increasing the survival of challenged brine shrimp larvae to over 80%. The only exception was 5-chloroindole, which also increased the survival of challenged brine shrimp larvae to over 80%. None of the iodinated indoles was able to increase the survival of challenged brine shrimp larvae to this level. Further, 2 out of the 3 multiple halogenated indoles did not improve the survival of challenged brine shrimp larvae, with the exception of 5-fluoro,6-chloroindole, which also increased the survival of challenged brine shrimp larvae to over 80%. Most of the halogenated indoles with another second substitution were not able to increase the survival of challenged brine shrimp larvae to over 80%, with the only three exceptions being those with a 3-carboxaldehyde substitution (i.e., analogues 10, 13, and 22). Finally, aminoindoles and nitroindoles did not protect brine shrimp from *V. campbellii* at the concentration that was tested.

**TABLE 2:** Published data of halogenated indole analogues and their impact on pathogens

Name	Structure	Phenotypic changes affected by indoles	Target pathogen	Reference
4-Fluoroindole	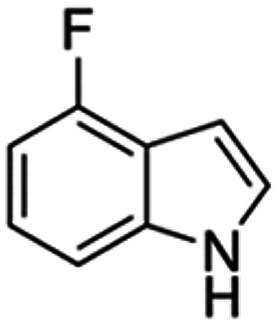	Inhibited biofilm formation	Candida albicans	47
5-Fluoroindole	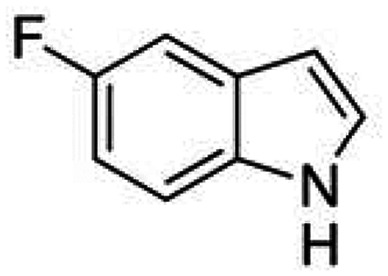	Inhibited QS of Chromobacterium violaceum CV026; inhibited prodigiosin production, biofilm formation, swimming motility, and swarming motility	Serratia marcescens	33
6-Fluoroindole	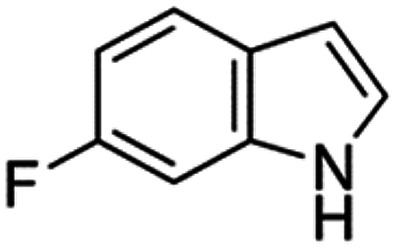	Inhibited QS of Chromobacterium violaceum CV026; inhibited prodigiosin production, biofilm formation, swimming motility, swarming motility, protease activity, and lipase activity	Serratia marcescens	33
7-Fluoroindole	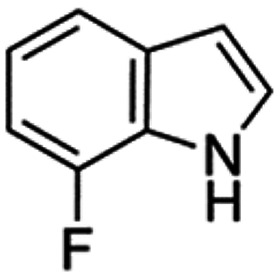	Inhibited biofilm formation, blood hemolysis of Pseudomonas aeruginosa; inhibited QS of Chromobacterium violaceum CV026; inhibited prodigiosin production, biofilm formation, swimming motility, and swarming motility of Serratia marcescens	Pseudomonas aeruginosa, Serratia marcescens	30, 33
4-Iodoindole	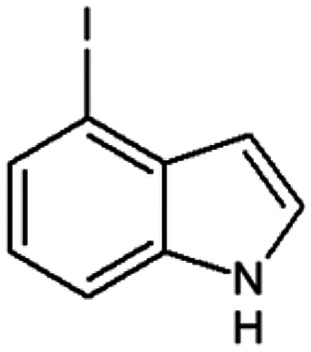	Inhibited biofilm formation, bacterial motility, fimbrial activity, hydrophobicity, protease activity, and indole production	Vibrio parahaemolyticus	29
5-Iodoindole	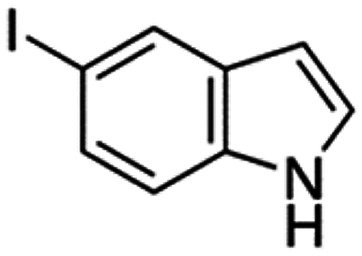	Inhibited biofilm formation of Candida albicans; inhibited prodigiosin production, biofilm formation, swimming motility, and swarming motility of Serratia marcescens; inhibited swimming motility, biofilm formation of Acinetobacter baumannii	Candida albicans, Serratia marcescens, Acinetobacter baumannii	47, 33, 50
6-Iodoindole	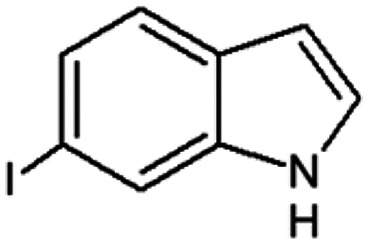	Inhibited biofilm formation, swimming motility, protease activity	Agrobacterium tumefaciens	48
7-Iodoindole	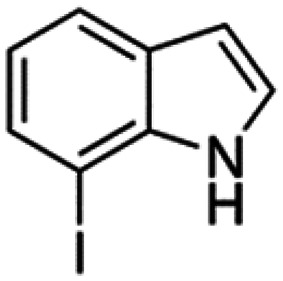	Inhibited biofilm formation, bacterial motility, fimbrial activity, hydrophobicity, protease activity, and indole production	Vibrio parahaemolyticus	29
4-Chloroindole	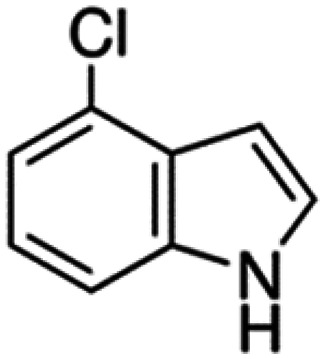	Inhibited biofilm formation, bacterial motility, fimbrial activity, hydrophobicity, protease activity, and indole production of V. parahaemolyticus; inhibited biofilm formation, swimming motility, protease activity of Agrobacterium tumefaciens; inhibited swarming and swimming motility and downregulated the expressions of virulence genes of Escherichia coli	Vibrio parahaemolyticus, Agrobacterium tumefaciens, Escherichia coli	29, 48, 49
5-Chloroindole	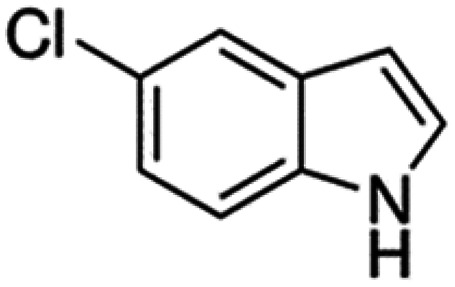	Inhibited swarming and swimming motility and downregulated the expressions of virulence genes of Escherichia coli	Escherichia coli	49
7-Chloroindole	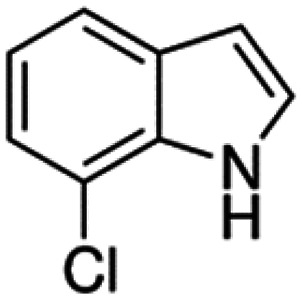	Inhibited biofilm formation, bacterial motility, fimbrial activity, hydrophobicity, protease activity, and indole production	Vibrio parahaemolyticus	29

**FIG 2:**
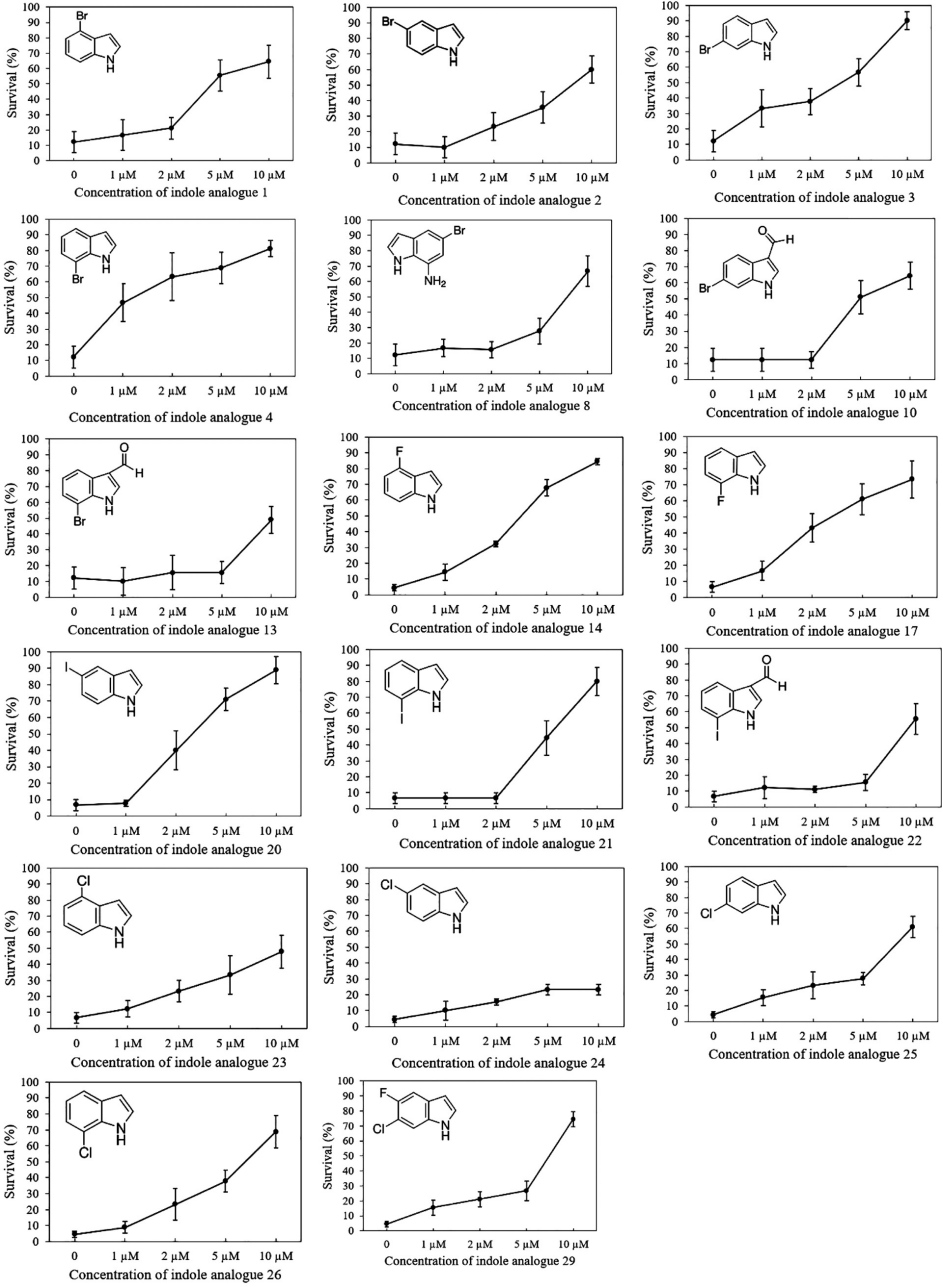
Percent survival of brine shrimp (Artemia franciscana) larvae after 2 days of challenge with Vibrio campbellii BB120 and different concentrations of selected indole analogues (average ± standard deviation of three shrimp cultures). The indole analogues were added to the brine shrimp rearing water at the start of the experiment. Error bars represent the standard deviation of three shrimp cultures. The survival of unchallenged larvae that were otherwise treated in the same way as challenged larvae was 98% ± 3%.

Seventeen indole analogues (i.e., indole analogues 1, 2, 3, 4, 8, 10, 13, 14, 17, 20, 21, 22, 23, 24, 25, 26, and 29, which increased the survival of challenged brine shrimp larvae to over 60% at 20 μM) were selected for more detailed experiments. The selected indole analogues were added into the brine shrimp rearing water at concentrations of 1, 2, 5, and 10 μM, respectively. After 2 days of culture, the survival of the brine shrimp larvae was found to be proportional to the concentration of the indole analogues (Fig. 2). Most of the selected indoles were able to increase the survival of challenged brine shrimp larvae to over 60% when added at 10 μM, except for analogues 13, 22, 23, and 24. The most active compounds increasing the survival of challenged brine shrimp to over 60% were 7-bromoindole (at 2 μM or more), 4-fluoroindole (5 μM), 7-fluoroindole (5 μM), and 5-iodoindole (5 μM). Five of the indoles were able to increase the survival of challenged brine shrimp larvae to over 80% (all at 10 μM) as follows: 6-bromoindole, 7-bromoindole, 4-fluoroindole, 5-iodoindole, and 7-iodoindole. The results of this second batch of brine shrimp challenge tests indicated that, in general, all of the compounds with a halogen (bromo, fluoro, iodo, or chloro) at position 7 are highly active in protecting brine shrimp larvae against *V. campbellii*. Several 7-substituted halogenated indole analogues, such as 7-fluoroindole, 7-iodoindole, and 7-chloroindole, have been reported before to affect bacterial virulence factors *in vitro* at higher concentrations than the active concentration found in this study. More specifically, 7-fluoroindole inhibited biofilm formation at a concentration of 500 μM and blood hemolysis at a concentration of 100 μM in Pseudomonas aeruginosa (30); 7-iodoindole and 7-chloroindole inhibited biofilm formation at a concentration of 10 μg/mL (about 40 to 60 μM), bacterial motility at a concentration of 50 μg/mL (about 200 to 300 μM), and protease activity at a concentration of 10 μg/mL in V. parahaemolyticus (29).

### Impact of selected indole analogues on swimming motility, biofilm formation, protease activity, and hemolytic activity of *V. campbellii*.

**FIG 3:**
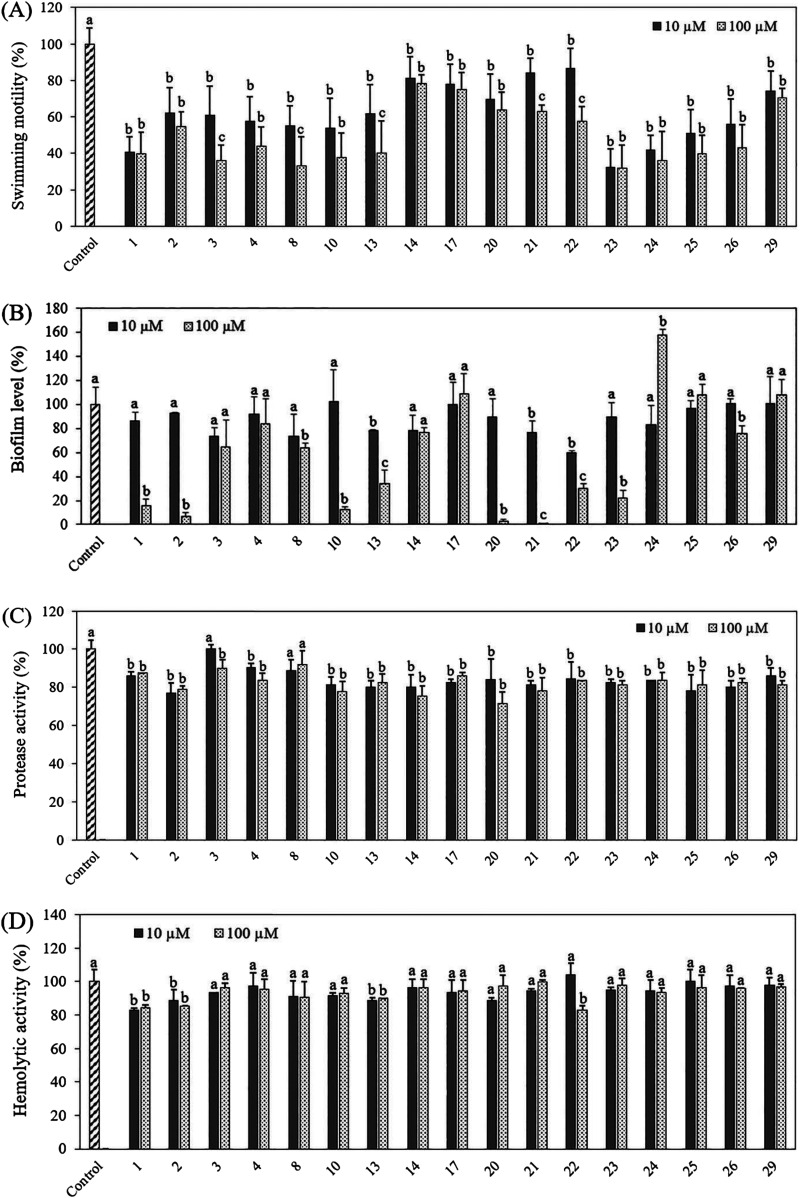
Impact of 10 and 100 μM selected indole analogues on swimming motility, biofilm formation, protease, and hemolytic activity of *V. campbellii* BB120. Data are presented as the mean ± standard deviation (SD) of six replicates for swimming motility and three independent experiments for biofilm formation, protease, and hemolytic activity. Control refers to untreated *V. campbellii.* The activities in this treatment were set at 100%, and other treatments were normalized accordingly (by multiplying with the same factor). For each analogue (each time comparing control, 10 μM, and 100 μM), different letters indicate significant differences in activity, whereas treatments receiving the same letter are not significantly different from each other (one-way analysis of variance [ANOVA] with Tukey’s *post hoc* test; *P* < 0.01).

Since the 17 selected indole analogues showed protective effect on brine shrimp larvae, further experiments were set up to explore how these indole analogues improved the survival by affecting the production of virulence factors of the pathogen, including swimming motility, biofilm formation, protease activity, and hemolytic activity. The results showed that all of the selected indole analogues decreased swimming motility of *V. campbellii* at both 10 μM and 100 μM (Fig. 3A). Brominated indoles (analogues 1, 2, 3, 4, 8, 10, and 13) and chlorinated indoles (analogues 23, 24, 25, and 26) showed stronger suppression of swimming motility than fluorinated, iodinated, and multiple halogenated indoles. Bacterial motility is viewed as a specific therapeutic target to cure or prevent disease, as it helps the cells colonize the host or abiotic surfaces (37, 38). Several halogenated indole analogues have also been reported before to decrease bacterial motility, including fluorinated indoles (5-fluoroindole, 6-fluoroindole, and 7-fluoroindole in Serratia marcescens), iodinated indoles (4-iodoindole and 7-iodoindole *in* V. parahaemolyticus and 5-iodoindole and 6-iodoindole in Acinetobacter baumannii), and chlorinated indoles (4-chloroindole and 5-chloroindole in Escherichia coli and 7-chloroindole in V. parahaemolyticus) (Table 2), which confirms that halogenated indole analogues have a significant effect on bacterial motility in different species of bacteria.

In the biofilm experiment, biofilm levels were decreased in the presence of indole analogues 1, 2, 8, 10, 13, 20, 21, 22, 23, and 26 at a concentration of 100 μM, while they were not affected at a concentration of 10 μM (Fig. 3B). Biofilm formation is important for the transmission of infections and the persistence of bacterial pathogens in hostile environments (39), and biofilm-forming *Vibrio* species are a major threat to the aquaculture industry (40). When in the biofilm state, bacteria are usually more resistant to antibiotics and disinfectants than planktonic bacteria (41). Therefore, it is desirable to select antimicrobial agents with the ability to decrease biofilm formation. It has been reported before that indole inhibited biofilm formation in *V. campbellii*, V. harveyi, and V. parahaemolyticus at a relatively high concentration of 200 μM (23), and 6-fluoroindole can inhibit bacterial biofilm formation in S. marcescens at a concentration of 250 μM (33). Interestingly, 4-fluoroindole and 7-fluoroindole were shown to decrease biofilm formation at lower concentrations of 20 μM and 100 μM in Candida albicans and S. marcescens (30, 42), whereas neither of them affected biofilm formation of *V. campbellii* in this study. Finally, 5-iodoindole, 7-iodoindole, 4-chloroindole, 5-chloroindole, and 7-chloroindole inhibited biofilm formation in C. albicans, V. parahaemolyticus, and E. coli at a concentration of 5 to 20 μg/mL (about 20 to 100 μM) (29, 42–44), which is consistent with the results obtained in this study.

In the protease activity and hemolytic activity experiments, the effect of selected indole analogues was smaller than in the swimming motility assay and the biofilm formation assay. Most selected indole analogues showed a small inhibitory effect on protease activity of *V. campbellii* at both 10 and 100 μM (except for analogues 3 and 8) (Fig. 3C). Most of the selected indole analogues did not affect the hemolytic activity of *V. campbellii* (Fig. 3D). As important virulence factors of pathogens, the impact of indole analogues on protease activity and hemolytic activity has also been explored before. It has been reported that 6-fluoroindole decreased protease activity of Serratia marcescens at a concentration of 500 μM (33). Further, 4-iodoindole, 7-iodoindole, 4-chloroindole, and 7-chloroindole inhibited protease activity of V. parahaemolyticus at a concentration of 10 to 100 μg/mL (about 40 to 600 μM) (29). Finally, 7-fluoroindole was shown to inhibit blood hemolysis without inhibiting the growth of Pseudomonas aeruginosa at a concentration of 100 μM (30). These results indicate that the impact of indoles on protease and hemolytic activities might be species dependent.

### Conclusions.

In conclusion, the present study demonstrated the abilities of halogenated indole analogues to protect brine shrimp larvae from pathogenic *V. campbellii* at a low concentration (≤20 μM) without affecting bacterial growth (no impact on growth curves up to 200 μM). This is consistent with the concept of antivirulence therapy, which does not kill the pathogens but rather blocks their virulence (15). We selected 17 halogenated indole analogues that improved the survival of brine shrimp larvae to over 60% at 20 μM and found that the most active compounds were 7-bromoindole (increasing the survival of challenged brine shrimp to over 60% at 2 μM or more), 4-fluoroindole (5 μM), 7-fluoroindole (5 μM), and 5-iodoindole (5 μM). Five of the indoles were able to increase the survival of challenged brine shrimp larvae to over 80% (all at 10 μM) as follows: 6-bromoindole, 7-bromoindole, 4-fluoroindole, 5-iodoindole, and 7-iodoindole. Further, indole analogues with another second substituent were found to work less well, and aminoindoles and nitroindoles did not affect the virulence of *V. campbellii*. Further *in vitro* work showed that all of the 17 selected halogenated indoles decreased swimming motility at both 10 μM and 100 μM, and most of them decreased biofilm formation at a concentration of 100 μM, whereas only a slightly decreased protease activity and no effect on hemolytic activity were observed. The fact that the compounds protected brine shrimp larvae at lower concentrations than those needed to inhibit the tested virulence factors suggests that the indole analogues also block a yet unknown virulence factor that is important for the virulence of *V. campbellii* to brine shrimp. However, further research will be needed to identify this virulence factor and to further establish the mode of action of the indole analogues.

## MATERIALS AND METHODS

### Bacterial strains, culture conditions, and chemicals.

Vibrio campbellii BB120 was used in this study. The bacteria were cultured in Luria-Bertani medium containing 35 g/L of sea salt (LB_35_) at 28°C under constant agitation (100 min^−1^). Cell densities were measured spectrophotometrically at 600 nm. Indole analogues were purchased from Sigma-Aldrich (Belgium) and are listed in Fig. 1. Each indole analogue was dissolved in dimethyl sulfoxide (DMSO) at 1, 2, 5, 10, 20, 100, and 200 mM, respectively. In all experiments, all treatments received the same volume of DMSO (0.1% vol/vol).

### Determination of the impact of the analogues on the growth curve of *V. campbellii*.

To investigate the effect of the halogenated indole analogues on the growth of *V. campbellii* BB120, overnight grown cells were inoculated into fresh LB_35_ medium at an initial optical density at 600 nm (OD_600_) of 0.01. Indole analogues were added at 0, 20, 100, or 200 μM, respectively. Then, 200-μL aliquots of these suspensions were pipetted into the wells of a polystyrene 96-well plate and cultured at 28°C for 24 h. The OD_600_ of each sample was measured by a Tecan Infinite M200 Pro plate reader every hour. Growth curves were determined for three independent cultures.

### Axenic hatching of brine shrimp larvae.

Five hundred milligrams of high-quality hatching cysts of Artemia franciscana (EGVR type; INVE Aquaculture, Baasrode, Belgium) were hydrated in 45 mL of filter-sterilized tap water for 1 h. Sterile cysts and larvae were obtained by decapsulation according to Marques et al. (45). In brief, 1.65 mL of NaOH (32%) and 25 mL of NaOCl (50%) were added to the hydrated cyst suspension to facilitate decapsulation. The process was stopped after 2 min by adding 35 mL of Na_2_S_2_O_3_ (10 g/L). Filtered (0.22 μm) aeration was provided during the reaction. The decapsulated cysts were washed with filtered and autoclaved artificial seawater (containing 35 g/L of instant ocean synthetic sea salt; Aquarium Systems, Sarrebourg, France). The cysts were resuspended in a bottle containing 1 L of filtered and autoclaved synthetic seawater and hatched for at least 28 h at 28°C with aeration and constant illumination (2,000 lx). The sterility of the cysts was verified by inoculating 1 mL of culture water into 9 mL of LB_35_ and incubating at 28°C for 24 h. After 28 h of hatching, batches of 30 larvae were counted and transferred into fresh, sterile 50-mL tubes containing 30 mL of filtered and autoclaved seawater. Finally, the tubes were put on a rotor (4 rotations per min) and kept at 28°C. All manipulations were performed in a laminar flow hood in order to maintain sterility of the cysts and larvae.

### Brine shrimp challenge tests.

The impact of indole analogues on the virulence of *V. campbellii* BB120 was determined using a standardized challenge test with gnotobiotic brine shrimp larvae as described by Defoirdt et al. (46). Briefly, *V. campbellii* BB120 was added into the sterile 50-mL tubes containing 30 mL of filtered and autoclaved seawater and 30 brine shrimp larvae at 10^6^ CFU/mL. The indole analogues (at the concentrations indicated in Results) were added into the brine shrimp rearing water at the start of the experiment. A suspension of autoclaved LVS3 bacteria (47) in filtered and autoclaved artificial seawater was added into all of the cultures as feed at the start of the challenge test at 10^7^ cells/mL. Brine shrimp cultures to which only DMSO and autoclaved LVS3 bacteria were added were used as controls. For each tube, the survival of the larvae was counted 48 h after the addition of the pathogen, and this value was converted to a percentage. Each treatment was carried out in triplicate. In each test, the sterility of the control treatments was checked at the end of the challenge by inoculating 1 mL of rearing water of the control treatment (no pathogens added) to 9 mL of LB_35_ and incubating the mixture for 2 days at 28°C. The concentrations of *V. campbellii* BB120 in the brine shrimp-rearing water at the end of the experiment were determined by plate counting on LB_35_ agar.

### Determination of swimming motility of *V. campbellii*.

The swimming motility assay was determined on LB_35_ soft agar plates containing 0.2% agar (48). Vibrio campbellii BB120 was grown overnight in LB_35_ medium and diluted to an OD_600_ of 1. The LB_35_ soft agar was cooled down to approximately 50°C after autoclaving. Then, the selected indole analogues were added at concentrations of 10 and 100 μM, respectively. The agar was poured into petri plates and left open at room temperature for 15 min. Five-microliter aliquots of the bacterial suspensions were added to the center of soft agar plates (6 replicate plates per treatment). The plates were incubated upright at 28°C, and the motility halo diameters were measured after 1 day.

### Determination of biofilm levels of *V. campbellii*.

Biofilm formation was quantified by crystal violet staining as described previously (49). Briefly, an overnight culture of *V. campbellii* BB120 was diluted to an OD_600_ of 0.01, and indole analogues were added at 10 and 100 μM. Then, 200-μL aliquots of these suspensions were pipetted into the wells of a polystyrene 96-well plate and cultured without agitation at 28°C for 24 h. After that, unattached cells were washed away with phosphate-buffered saline (PBS) three times. Subsequently, the remaining attached bacteria were fixed with 200 μL methanol per well for 20 min, after which the methanol was removed, and the plates were air-dried. Then, biofilms were stained for 15 min with 200 μL per well of a 1% crystal violet solution. Plates were then rinsed with tap water to remove nonadherent cells by three times. After the plates were air-dried, bound crystal violet was dissolved in 200 μL of 95% ethanol per well for 30 min, and absorbance was measured at 570 nm with a Tecan Infinite M200 Pro plate reader. Sterile medium served as a negative control, and the reported values are blank-corrected.

### Determination of protease and hemolytic activity of *V. campbellii*.

Protease and hemolytic activity assays were performed according to Natrah et al. (50) with some modifications. The protease assay plates were prepared by mixing double strength LB_35_ agar (30g/L) with a 4% skim milk powder suspension (Oxoid, Basingstoke, Hampshire, UK) sterilized separately at 121°C for 5 min. Hemolytic assay plates were prepared by supplementing LB_35_ agar with 5% defibrinated sheep blood (Oxoid). The selected indole analogues were added into agar before it was poured into petri plates at concentrations of 10 and 100 μM, respectively. All agar plates were opened at room temperature for 15 min to dry. Then the bacterial suspension was applied to the surface of an agar plate (at least 3 replicates per treatment). The plates were incubated upright at 28°C, and the clearing zone diameter and colony diameter were measured after 4 days of incubation.
