# Supplementary material for: Halogenated Indoles Decrease the Virulence of Vibrio campbellii in a Gnotobiotic Brine Shrimp Model
Source: Microbiol Spectr. 2022 Sep 26;10(5):e02689-22. doi: 10.1128/spectrum.02689-22 (PMC9602911; doi:10.1128/spectrum.02689-22)
Supplement: Supplemental file 1 — Supplemental material. Download spectrum.02689-22-s0001.pdf, PDF file, 2.0 MB [file spectrum.02689-22-s0001.pdf]

Halogenated indoles decrease the virulence of *Vibrio campbellii* in a gnotobiotic brine shrimp model – supplemental material

Shanshan Zhang, Qian Yang, Tom Defoirdt

Figure S1. Impact of the indole analogues on the growth of *Vibrio campbellii* BB120 in LB_{35} medium. Growth was measured spectrophotometrically at 600 nm.

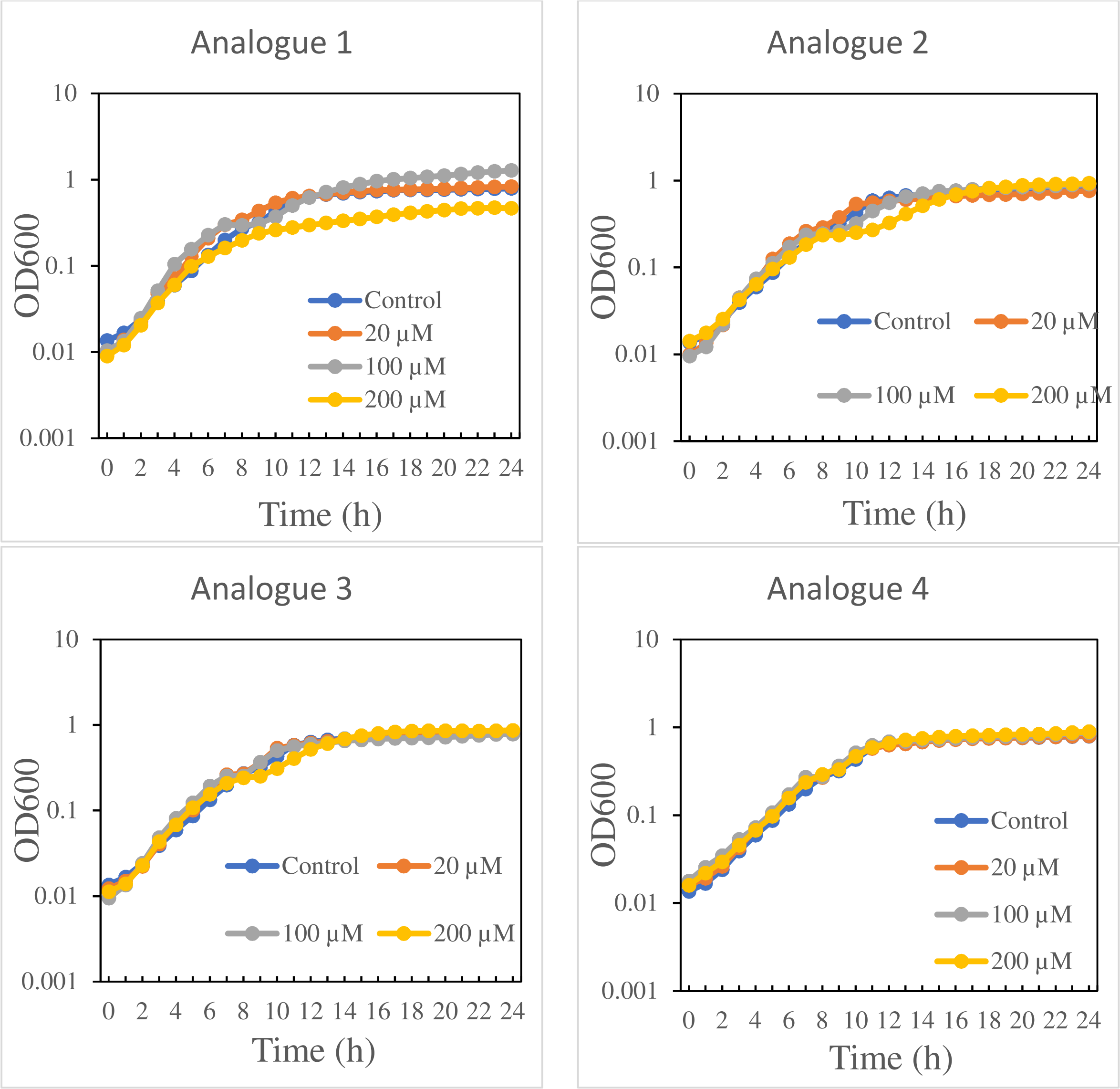

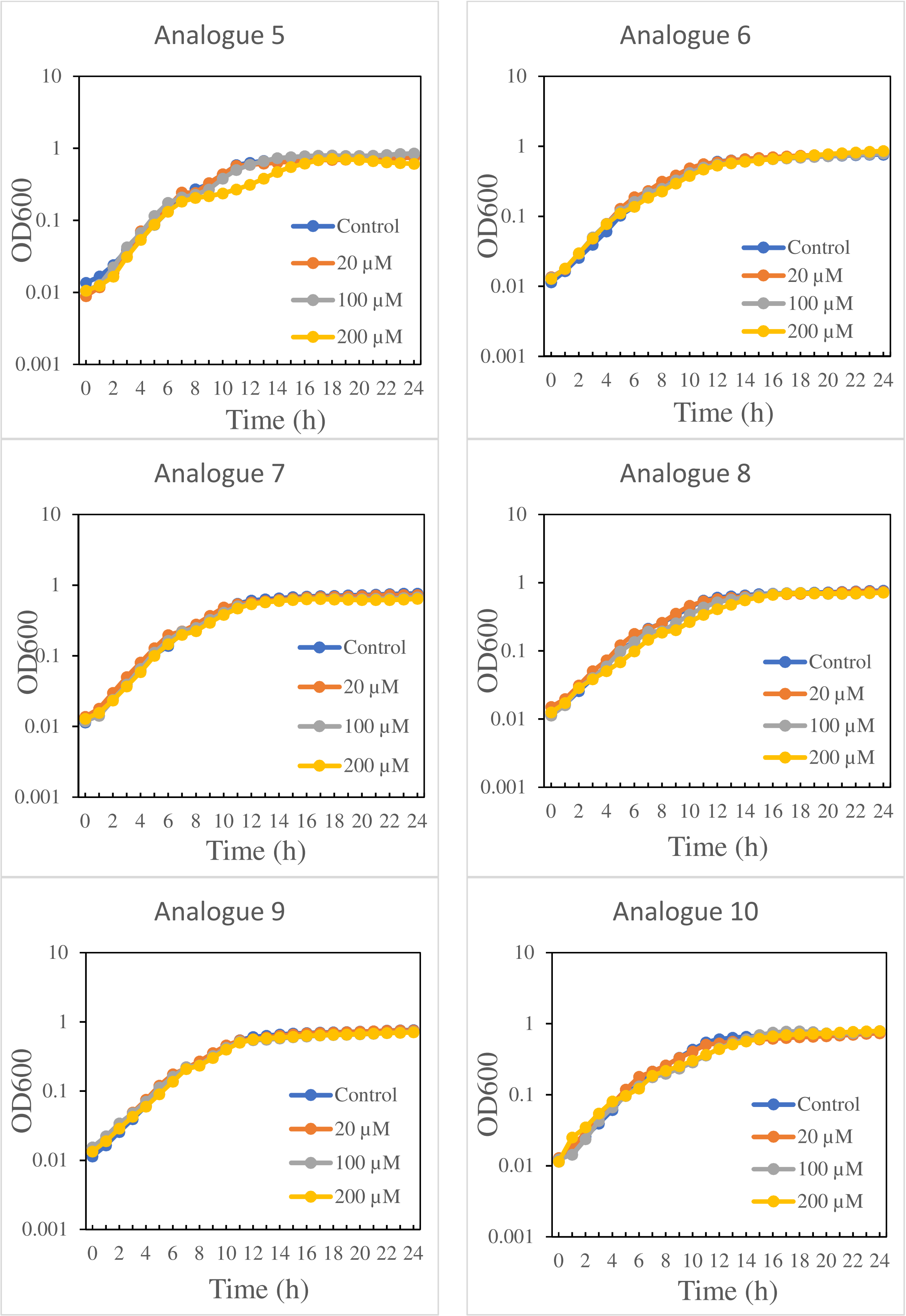
Analogue 5
OD600
Time (h)
Control
20 µM
100 µM
200 µM
Analogue 6
OD600
Time (h)
Control
20 µM
100 µM
200 µM
Analogue 7
OD600
Time (h)
Control
20 µM
100 µM
200 µM
Analogue 8
OD600
Time (h)
Control
20 µM
100 µM
200 µM
Analogue 9
OD600
Time (h)
Control
20 µM
100 µM
200 µM
Analogue 10
OD600
Time (h)
Control
20 µM
100 µM
200 µM

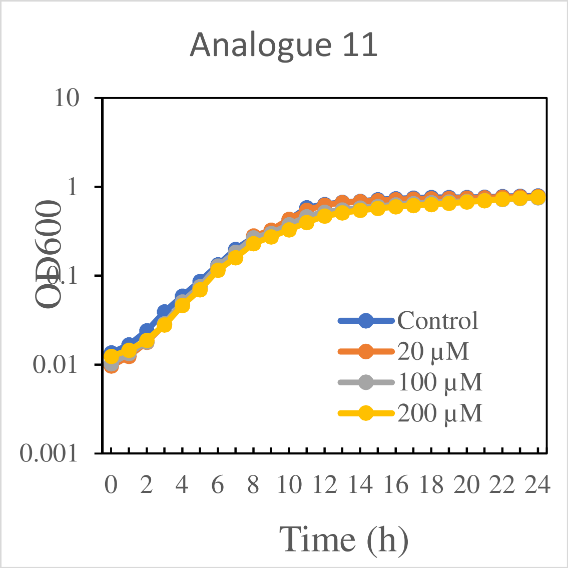
Analogue 11
OD600
10
1
0.1
0.01
0.001
0 2 4 6 8 10 12 14 16 18 20 22 24
Time (h)
Control
20 µM
100 µM
200 µM

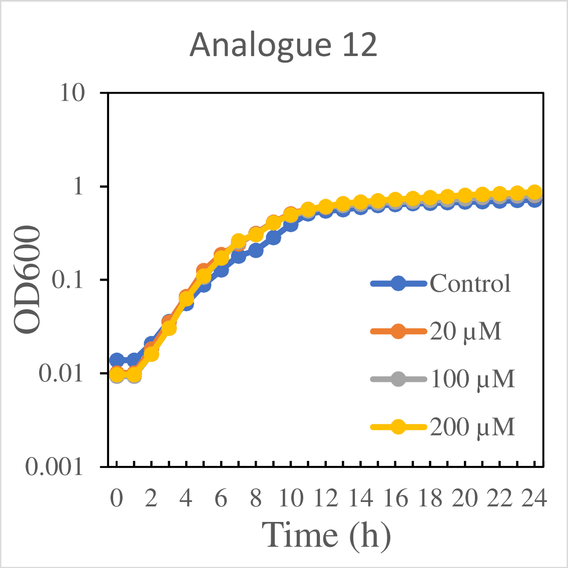
Analogue 12
OD600
10
1
0.1
0.01
0.001
0 2 4 6 8 10 12 14 16 18 20 22 24
Time (h)
Control
20 µM
100 µM
200 µM

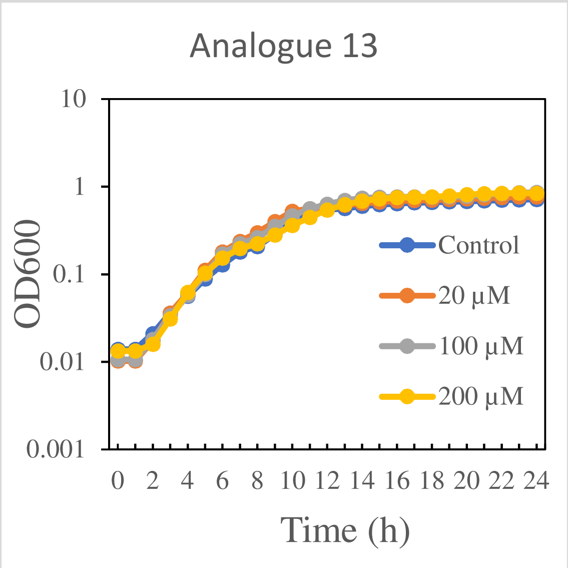
Analogue 13
OD600
10
1
0.1
0.01
0.001
0 2 4 6 8 10 12 14 16 18 20 22 24
Time (h)
Control
20 µM
100 µM
200 µM

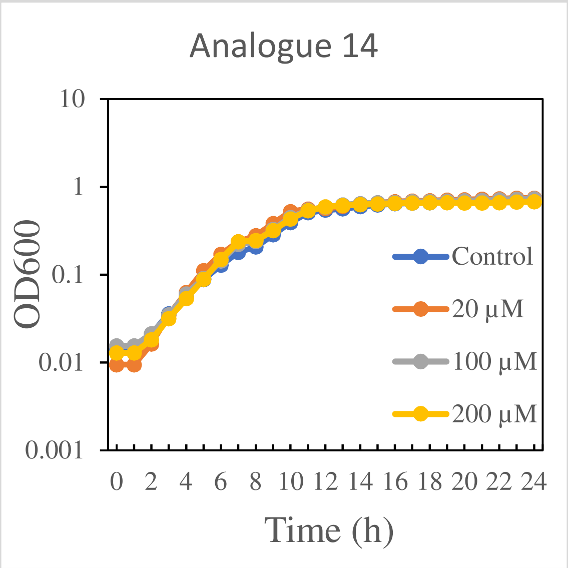
Analogue 14
OD600
10
1
0.1
0.01
0.001
0 2 4 6 8 10 12 14 16 18 20 22 24
Time (h)
Control
20 µM
100 µM
200 µM

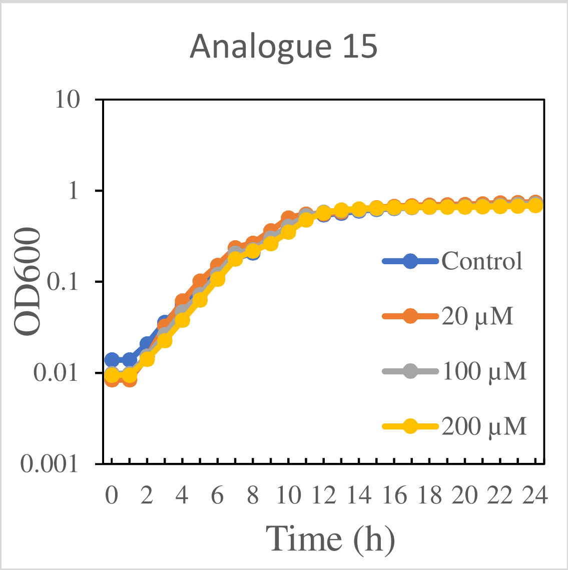
Analogue 15
OD600
10
1
0.1
0.01
0.001
0 2 4 6 8 10 12 14 16 18 20 22 24
Time (h)
Control
20 µM
100 µM
200 µM

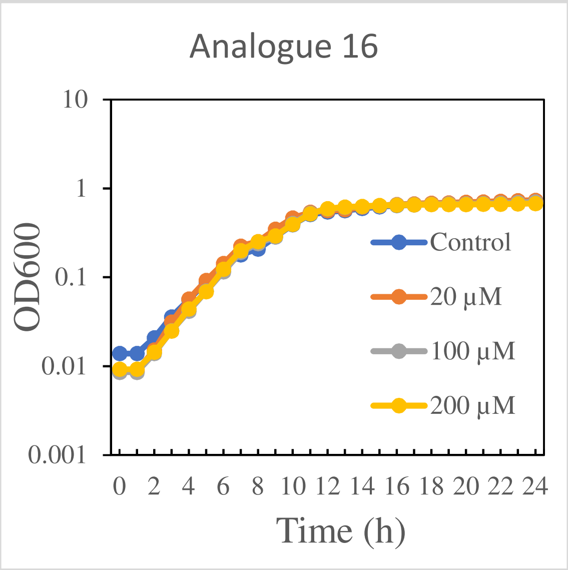
Analogue 16
OD600
10
1
0.1
0.01
0.001
0 2 4 6 8 10 12 14 16 18 20 22 24
Time (h)
Control
20 µM
100 µM
200 µM

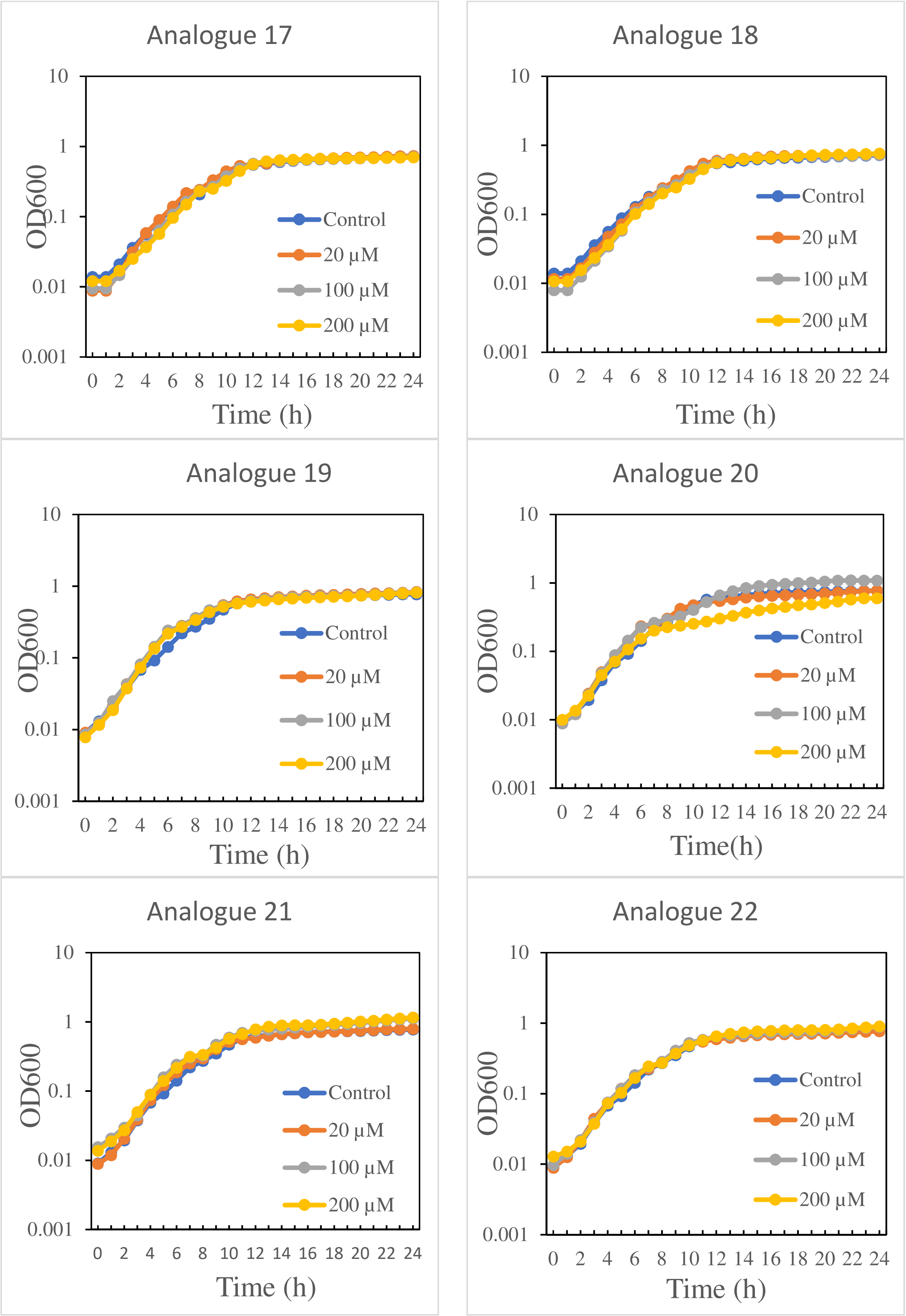
Analogue 17
Analogue 18
Analogue 19
Analogue 20
Analogue 21
Analogue 22
OD600
10
1
0.1
0.01
0.001
0 2 4 6 8 10 12 14 16 18 20 22 24
Time (h)
Time(h)
Control
20 µM
100 µM
200 µM

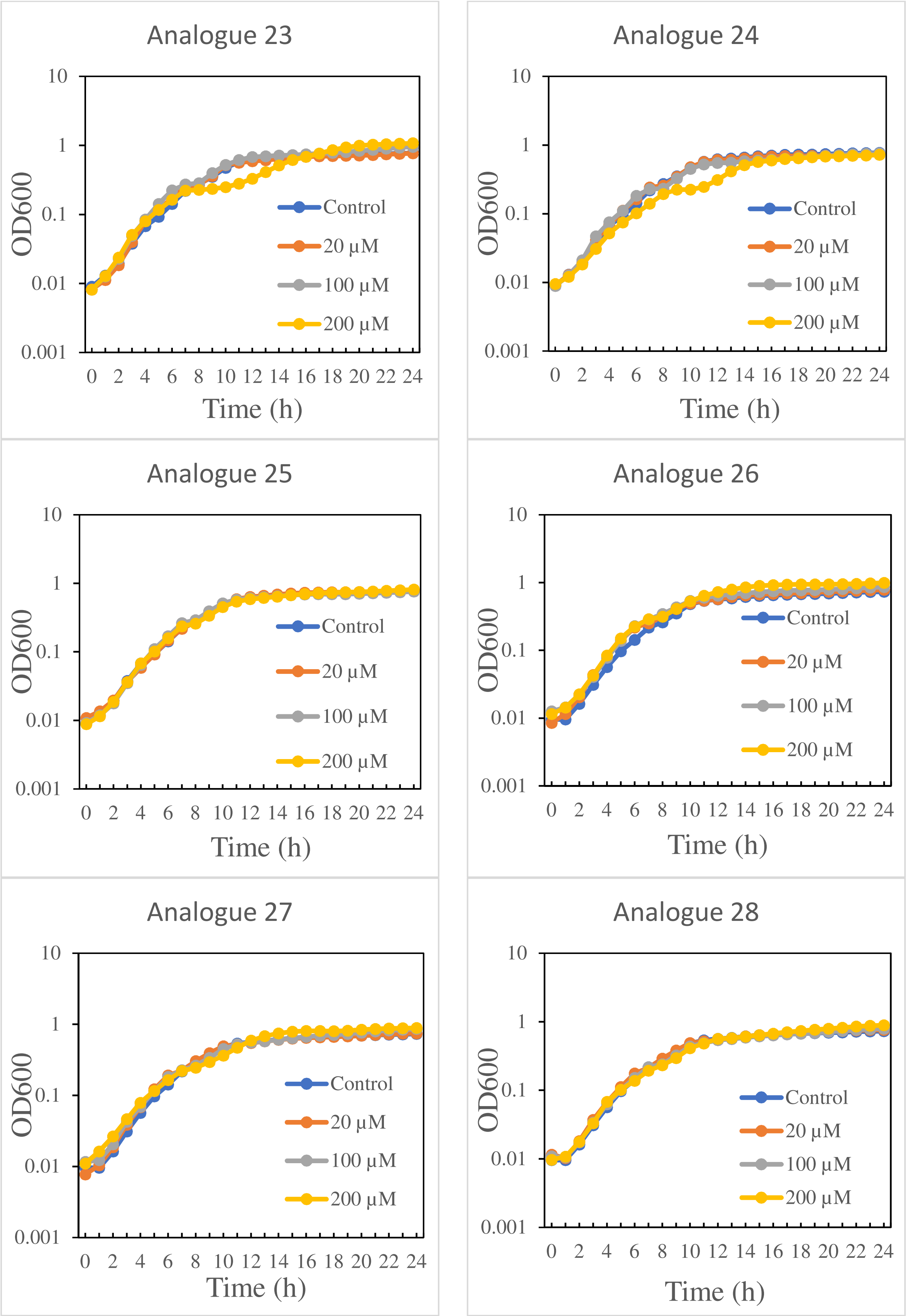
Analogue 23
Analogue 24
Analogue 25
Analogue 26
Analogue 27
Analogue 28
OD600
Time (h)
10
1
0.1
0.01
0.001
0 2 4 6 8 10 12 14 16 18 20 22 24
Control
20 µM
100 µM
200 µM

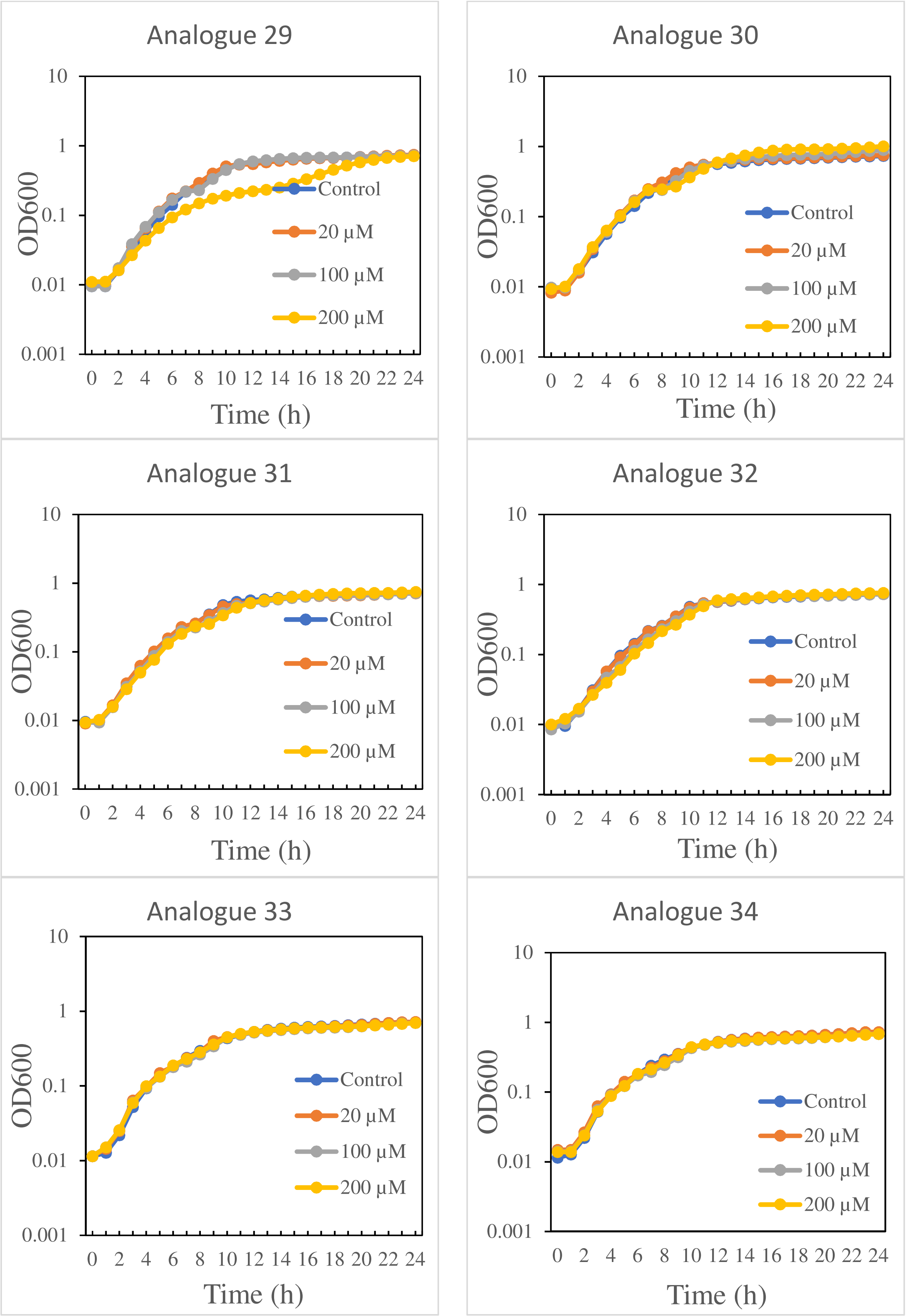
Analogue 29
Analogue 30
Analogue 31
Analogue 32
Analogue 33
Analogue 34
OD600
10
1
0.1
0.01
0.001
0 2 4 6 8 10 12 14 16 18 20 22 24
Time (h)
Control
20 µM
100 µM
200 µM

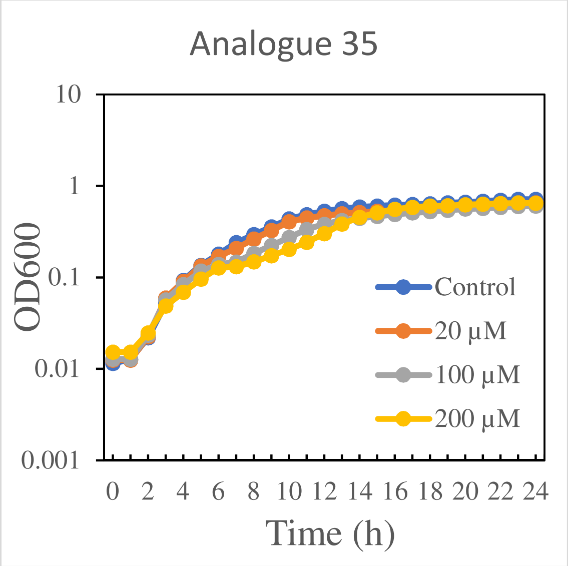
Analogue 35
OD600
10
1
0.1
0.01
0.001
0 2 4 6 8 10 12 14 16 18 20 22 24
Time (h)
Control
20 µM
100 µM
200 µM

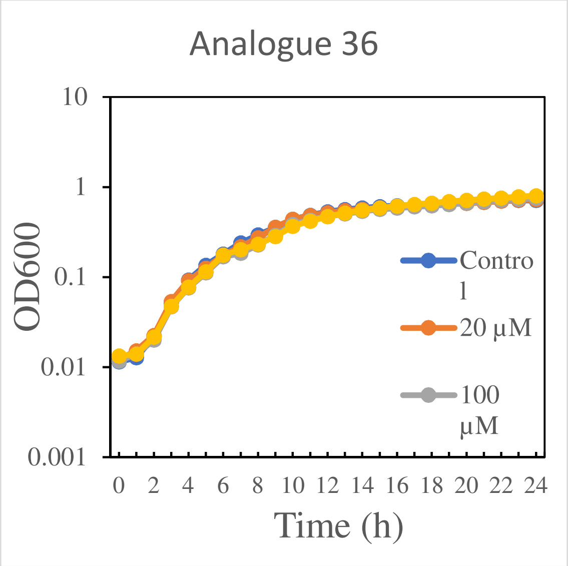
Analogue 36
OD600
10
1
0.1
0.01
0.001
0 2 4 6 8 10 12 14 16 18 20 22 24
Time (h)
Contro
l
20 µM
100
µM

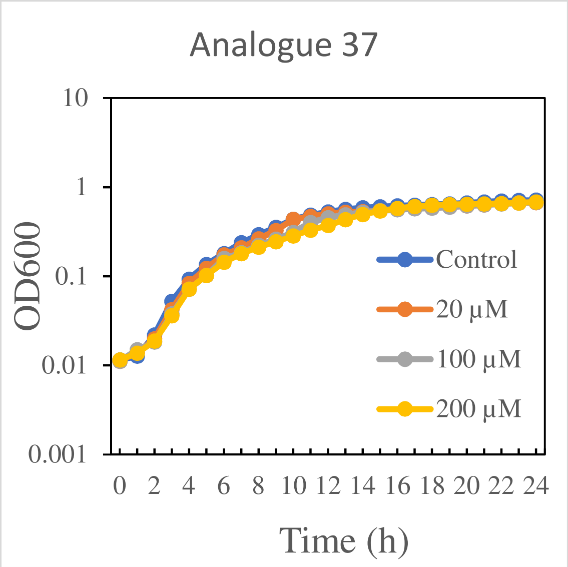
Analogue 37
OD600
10
1
0.1
0.01
0.001
0 2 4 6 8 10 12 14 16 18 20 22 24
Time (h)
Control
20 µM
100 µM
200 µM

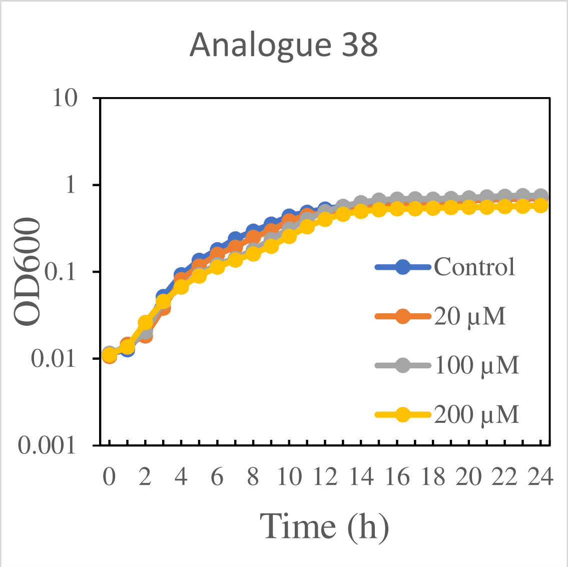
Analogue 38
OD600
10
1
0.1
0.01
0.001
0 2 4 6 8 10 12 14 16 18 20 22 24
Time (h)
Control
20 µM
100 µM
200 µM

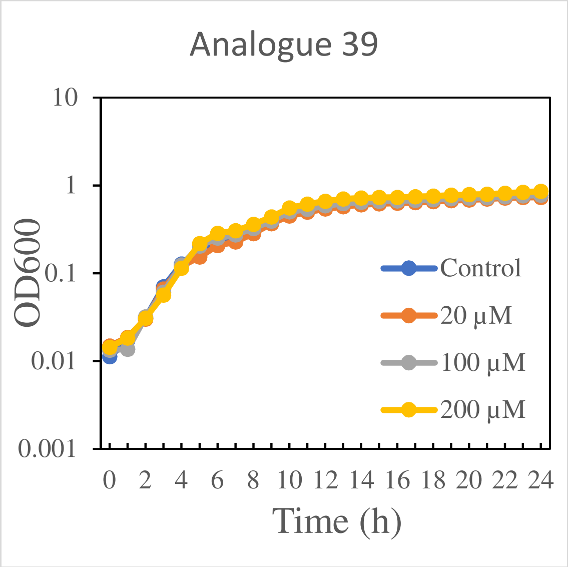
Analogue 39
OD600
10
1
0.1
0.01
0.001
0 2 4 6 8 10 12 14 16 18 20 22 24
Time (h)
Control
20 µM
100 µM
200 µM

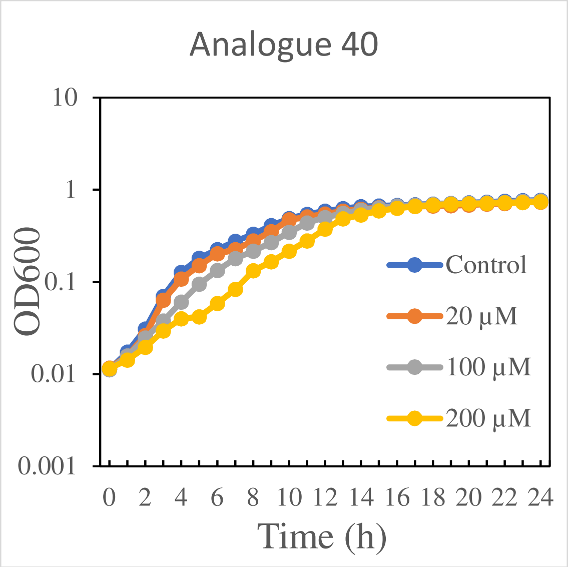
Analogue 40
OD600
10
1
0.1
0.01
0.001
0 2 4 6 8 10 12 14 16 18 20 22 24
Time (h)
Control
20 µM
100 µM
200 µM

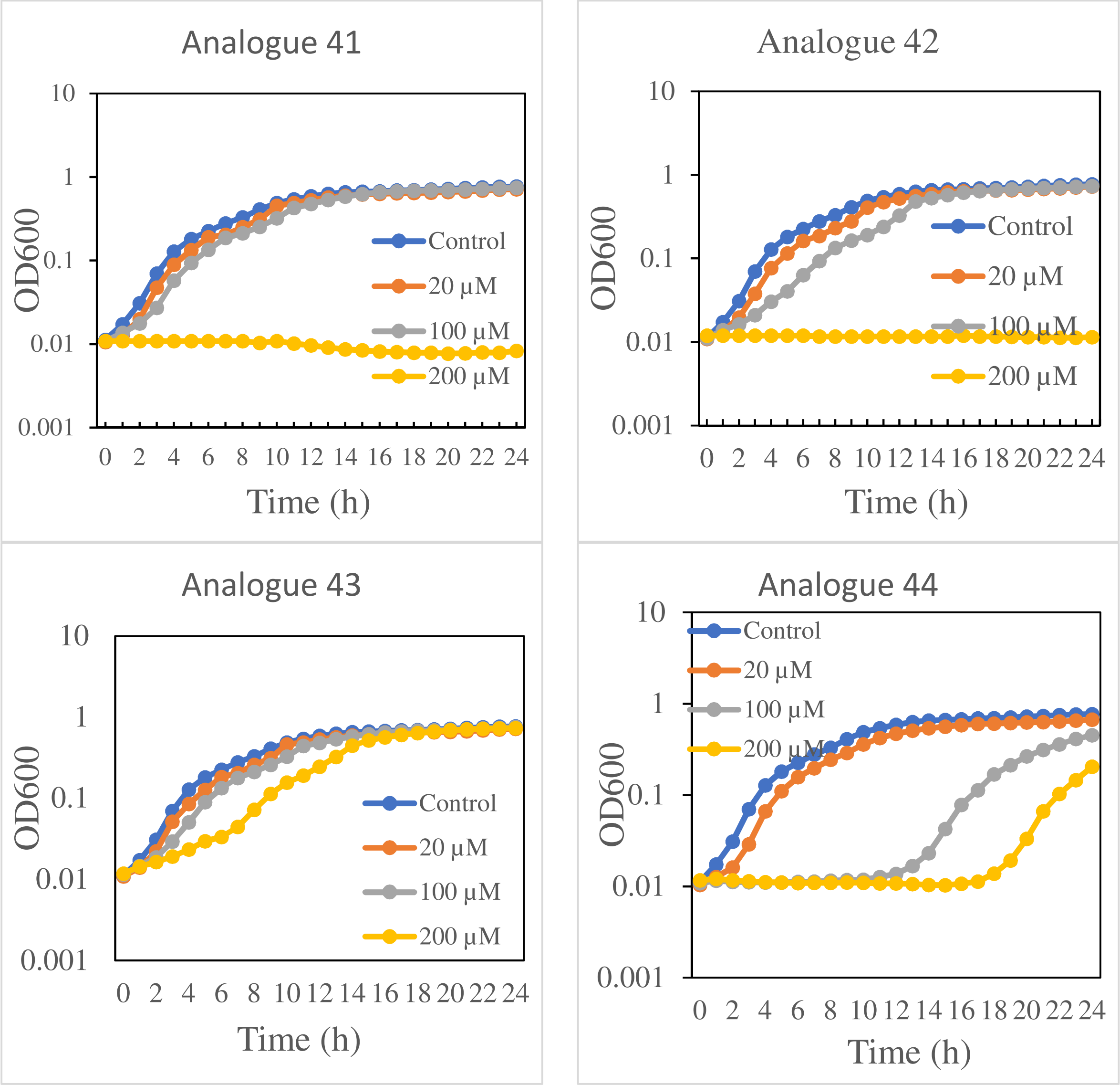
Analogue 41
OD600
Time (h)
Control
20 µM
100 µM
200 µM
Analogue 42
OD600
Time (h)
Control
20 µM
100 µM
200 µM
Analogue 43
OD600
Time (h)
Control
20 µM
100 µM
200 µM
Analogue 44
OD600
Time (h)
Control
20 µM
100 µM
200 µM
